# IL-13 receptors as possible therapeutic targets in diffuse intrinsic pontine glioma

**DOI:** 10.1371/journal.pone.0193565

**Published:** 2018-04-05

**Authors:** Noah E. Berlow, Matthew N. Svalina, Michael J. Quist, Teagan P. Settelmeyer, Viktor Zherebitskiy, Mari Kogiso, Lin Qi, Yuchen Du, Cynthia E. Hawkins, Esther Hulleman, Xiao-Nan Li, Sakir H. Gultekin, Charles Keller

**Affiliations:** 1 Children's Cancer Therapy Development Institute, Beaverton, OR, United States of America; 2 Department of Pathology, Oregon Health & Science University, Portland, OR, United States of America; 3 Department of Pediatrics, Texas Children's Cancer Center, Houston, TX, United States of America; 4 Division of Pathology, The Hospital for Sick Children, Toronto, ON, CANADA; 5 Neuro-Oncology Research Group, Cancer Center Amsterdam, Vrije Universiteit University Medical Center, Amsterdam, The Netherlands; 6 Department of Pediatrics, Oregon Health & Science University, Portland, OR, United States of America; Northwestern University, UNITED STATES

## Abstract

Diffuse intrinsic pontine glioma (DIPG) is a universally fatal childhood cancer of the brain. Despite the introduction of conventional chemotherapy and radiotherapy, improvements in survival have been marginal and long-term survivorship is uncommon. Thus, new targets for therapeutics are critically needed. Early phase clinical trials exploring molecularly-targeted therapies against the epidermal growth factor receptor (*EGFR*) and novel immunotherapies targeting interleukin receptor-13α2 (*IL-13Rα2*) have demonstrated activity in this disease. To identify additional therapeutic markers for cell surface receptors, we performed exome sequencing (16 new samples, 22 previously published samples, total 38 with 26 matched normal DNA samples), RNA deep sequencing (17 new samples, 11 previously published samples, total 28 with 18 matched normal RNA samples), and immunohistochemistry (17 DIPG tissue samples) to examine the expression of the interleukin-4 (IL-4) signaling axis components (*IL-4*, interleukin 13 (*IL-13*), and their respective receptors *IL-4Rα*, *IL-13Rα1*, and *IL-13Rα2)*. In addition, we correlated cytokine and receptor expression with expression of the oncogenes *EGFR* and *c-MET*. In DIPG tissues, transcript-level analysis found significant expression of *IL-4*, *IL-13*, and *IL-13Rα1/2*, with strong differential expression of *IL-13Rα1/2* in tumor versus normal brain. At the protein level, immunohistochemical studies revealed high content of *IL-4* and *IL-13Rα1/2* but notably low expression of *IL-13*. Additionally, a strong positive correlation was observed between *c-Met* and *IL-4Rα*. The genomic and transcriptional landscape across all samples was also summarized. These data create a foundation for the design of potential new immunotherapies targeting IL-13 cell surface receptors in DIPG.

## Introduction

Brainstem gliomas account for up to 20% of all central nervous system tumors in children less than 15 years of age, with a median age at presentation of 6–7 years[1]. Diffuse intrinsic brainstem gliomas (DIPG) comprise 80% of all brainstem gliomas and are typically anaplastic astrocytoma (AA), glioblastoma multiforme (GBM) or Grade 2 lesions[2]. No trials have yet shown benefit of chemotherapy for management of patients with diffuse intrinsic pontine glioma[3]. In the United States, approximately 350 new patients per year are diagnosed with DIPG. Prognosis for patients with DIPG is poor, with a median survival of less than 1 year; fewer than 20% of children are alive at 2 years[4]. Standard therapy consists of conventional local field radiotherapy to a dose of 54–60 Gy for 6 weeks. Without radiotherapy, median survival is approximately 20 weeks[1, 5]. Radiotherapy leads to transient improvements in neurological function and improves overall survival by approximately 2–3 months[6]. Multiple studies using a variety of conventional, high-dose and targeted therapies have demonstrated no survival advantage in patients with DIPG [7–13]. Thus, novel therapies are clearly needed for patients with DIPG.

Early phase clinical trials evaluating the safety and efficacy of the *EGFR* inhibitors gefitinib and erlotinib have demonstrated modest activity in brainstem gliomas similar to DIPG[14]. Although many previous small molecule inhibitors have demonstrated no significant benefit, an expanding understanding of the mutational landscape and defined therapeutic targets have led us to discoveries of a set of potentially effective small molecule inhibitors. Recently published work from our group prioritized panobinostat[15], an epigenome-modifying small molecule histone deacetylase, via *in vitro* and *in vivo* validation on DIPG primary cell cultures.

An alternative approach focuses on antibody drug conjugates (ADCs); the emergence of bivalent antibodies makes targeting both receptors and receptor tyrosine kinases in DIPG a viable therapeutic avenue. Based on our newly-generated data, *IL-4*, *IL-13* and their receptors *IL-4Rα*, *IL-13Rα1*, *IL-13Rα2* may be reasonable therapeutic targets in DIPG due to differential overexpression on the cell surface of cancerous cells. This is consistent with previous research showing the potential of *IL-4* and *IL-13Rα2* as potential therapeutic targets in pediatric brain tumors such as glioblastoma [16–22].

*IL-4* and *IL-13* tethered exotoxins have already led to clinical trials, *e*.*g*., the *IL-13*PE38QQR trial NCT00880061 at NIH[16, 23] and the *IL-4*::Pseudomonas Exotoxin product PRX321 in development by Protox Therapeutics as NCT00797940[24]. Similarly, *IL-13Rα2* as an immunogen is the basis of a DIPG/high grade glioma vaccine trial [NCT01130077]. This pilot clinical trial evaluated the safety and efficacy of subcutaneous vaccination with glioma-associated antigens (GAAs) and their epitope peptides including *IL-13Rα2*, *EphA2*, and *Survivin*. Five of twenty-six patients experienced inflammatory-associated pseudoprogression, defined as six-month progression-free survival following transient clinical progression, with one patient experiencing an event free survival of greater than three years [25, 26]. Thus, targeting overexpressed glioma-specific receptors or receptor tyrosine kinases in combination with targeted antibodies may further improve survival. A case report of *IL13RA2*-targeted chimeric antigen receptor T-cell therapy showed regression of glioblastoma in a human patient[27]. Dual receptor targeting of *EGFR* and *c-Met*, possible using certain bivalent antibody approaches [28–30], may be reasonable combination therapeutic targets in high grade glial tumors based on expression studies [31–35] from which one DIPG clinical trial employing the *EGFR* inhibitor, erlotinib, has opened [NCT01182350].

Based on the newly-generated sequencing data, *IL-4* and *IL-13* axis components were identified as being targets of interest in DIPG. Thus, in this work we explore *IL-4* or *IL-13* axis components as well as EGFR or c-MET as combination therapy targets in DIPG. We have surveyed archived human DIPG cases (28 by RNA-seq, 17 by immunohistochemistry (IHC)) for *IL-4*, *IL-13*, *IL-4Rα*, *IL-13Rα1*, *IL-13Rα2*, *EGFR* and *c-Met* expression. The studies herein demonstrate that most DIPG tissues displayed a high content of *IL-4* and weak or absent expression of *IL-13*, yet modest but significant expression of *IL-4* and *IL-13* was observed at the transcript level. By contrast, *IL-13Rα1* and *IL-13Rα2* were found to be significantly enriched at the transcript level in DIPG tissues and expression by IHC was found to be high with 41% of these tissues also staining positive for the oncogenes *EGFR* and *c-Met*.

## Results

### Comparison of gene expression in DIPG tissues with matched normal samples

**Fig 1:**
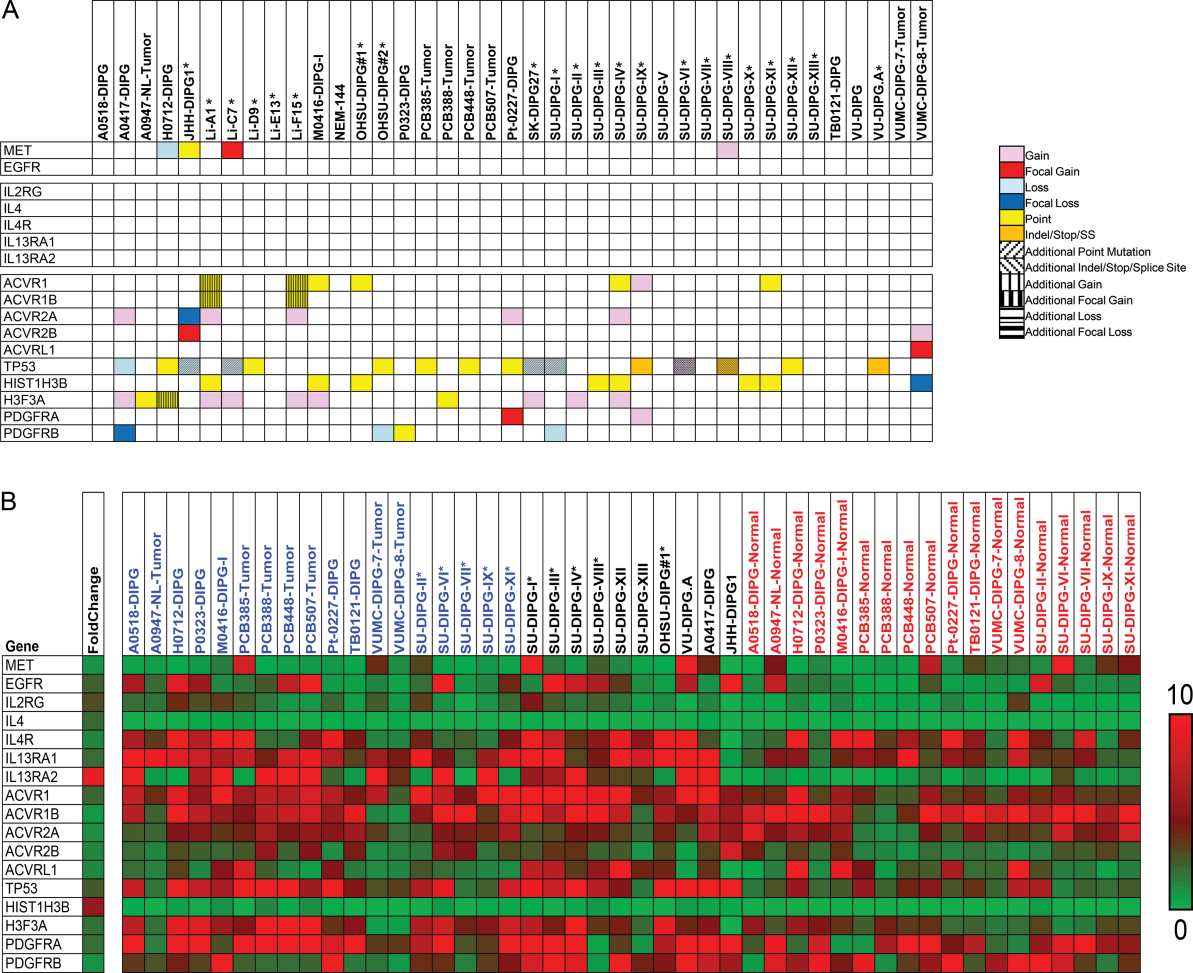
Landscape of exome mutations and differential gene expression of frequently-mutated DIPG genes and IL-4 pathway targets in DIPG samples. (A) Mutational landscape of frequently-mutated DIPG genes and IL-family genes. Exome sequencing was performed on 38 DIPG samples to identify somatic mutations and copy number variations. When possible, tumor samples were compared against matched normal samples. (B) Gene expression of frequently-mutated DIPG genes and IL-family genes. The set of genes is identical to those in Fig A. RNA sequencing was performed on 28 DIPG samples: 18 tumor samples with matched normal samples, 10 unmatched tumor samples. Average fold change and tumor vs. normal p-values were calculated across the set of expressed genes. Note that *IL-13Rα1* and *IL-13Rα2* are significantly overexpressed in tumor compared to normal, but are mutationally silent.

To characterize the mutation status and gene expression levels of the cytokines *IL-4* and *IL-13*, their receptors *IL-4Rα*, *IL-13Rα1/2*, and the oncogenes *c-Met* and *EGFR*, we performed exome and RNA-seq analysis on DIPG tissues (38 tumor exome, 26 normal exome, 28 tumor RNA, 18 normal RNA, Fig 1,S1 and S2 Figs). Exome sequencing did not identify any somatic point mutations or copy number variations in *IL-4* signaling axis genes. At the transcript level, we observed statistically significant differential gene expression in *IL-4* (p = 0.035), *IL-13* (p = 0.016), *IL-13Rα1* (p = 5.3e^-3^), and *IL-13Rα2* (p = 8.0e^-4^). *IL-13Rα1* (2.02-fold overexpression, p = 0.0007) and *IL-13Rα2* (17.32-fold overexpression, p = 0.0003) were statistically differentially overexpressed in DIPG samples, with *IL-13Rα2* overexpression notably higher. No statistically significant differential expression was observed in *EGFR* (p = 0.68), *c-Met* (p = 0.23), or *IL-4Rα* (p = 0.88) (Fig 1B). Statistical analysis revealed a significant correlation between expression of c-Met and both *IL-4Rα* (r = 0.734, p < 0.0001) and *IL-13Rα1*(r = 0.5325, p = 0.004, respectively); correlation was also observed between *EGFR* and *IL-13Rα2* (r = 0.3865, p = 0.04) (Table 1 and Table 2).

**Table 1:** Summary of immunohistochemical staining.

Antibody	Positive	M/S Labeling	Score 2 or 3	Negative	Not Performed	# of cases
*IL-4*	12	6/12	9/12	2	3	17
*IL-4R*	12	5/12	9/12	3	2	17
*IL-13*	1	0/1	0/1	13	3	17
*IL-13A1*	13	5/13	10/13	3	1	17
*IL-13A2*	12	6/12	6/12	4	1	17
*EGFR*	7	5/7	5/7	8	2	17
*MET*	7	5/7	5/7	7	3	17

For Table 1, staining definitions are as follows: 0 = no labeling, 1 = < 10%, 2 = 10–50%, 3 = >50%; W = Weak, M = Medium, S = Strong.

**Table 2:** EGFR correlation with IL-4 signaling axis genes and correlation between IL-4 tumor and normal.

	*EGFR* vs. *IL-4*	*EGFR* vs. *IL-4R*	*EGFR* vs. *IL-13*	*EGFR* vs. *IL-13Rα1*	*EGFR* vs. *IL-13Rα2*	*IL-4R Tu* vs. *IL-4R Nm*
R squared	0.1162	0.0176	0.0984	0.0065	0.1494	0.2718
P value (two-tailed)	0.0818	0.5092	0.111	0.6905	**0.0464**	**0.0265**
# of paired samples	27	27	27	2	27	18

RNA-seq identified 3,577 (11.6% of 30,796 genes) differentially upregulated and 3,365 (10.9% of 30,796 genes) differentially downregulated genes in tumor samples relative to normal samples. Among genes frequently mutated (defined as 7 or more variations in that gene across the 38 sequenced samples), 8 genes were significantly downregulated (p < 0.01) in tumor samples (*CAMK1G*, *MINK1*, *PARD6A*, *CDC42BPA*, *CDC42BPB*, *MAP3K9*, *RPS6KA5*) and 17 genes were significantly upregulated (p < 0.01) in tumor samples (*MYC*, *ACVR1*, *NTRK1*, *MAPKAPK2*, *IL-13RA1*, *IL-13RA2*, *TP53*, *CLK2*, *HIST1H3B*, *ABL2*, *IL2RG*, *DDR2*, *MAPK7*, *PSENEN*, *STK36*, *H3F3A*, *IKBKE*). Several genes located in the 1q cytogenetic band (*DDR2*, *HSPA6*, *MAP3K9*, *ABL2*, *CDK18*, *IKBKE*, *MDM4*, *NUAK2*, *PIK3C2B*, *CAMK1G*, *NEK2*, *MARK1*, *PARP1*, *CDC42BPA*, *AKT3*) were noted to have gain events in several samples. Frequent 1q gain events have been associated with poor prognosis in a variety of pediatric diseases including neuroblastoma and medulloblastoma[36]. This analysis was generated solely from the Illumina sequencing data, and may not completely capture the mutational landscape of these DIPG samples due to coverage limitations of high throughput sequencing technology. A complete table of genomic variants is provided in S1 Table, and a complete table of gene expression data is provided in S2 Table.

### Key mutations identified by Sanger sequencing

Sanger sequencing was performed on VUMC-DIPG-7 and VUMC-DIPG-8 samples independently of the main body of research. Sanger sequencing identified *H3F3A* (K27M) mutations (Fig 1A), consistent with other DIPG samples[37]. In previously reported samples, 6/16 (37.5%) samples carry *H3F3A* mutations; in new samples for this report, 5/22 (22.7%) carry *H3F3A* mutations. *H3F3A* mutations were not identified in the Illumina sequencing due to lack of coverage at the *H3F3A* loci; nonetheless, coverage for other regions was sufficient to identify mutations in other genes.

### Immunohistochemical studies of the IL-4R signaling axis

**Fig 2:**
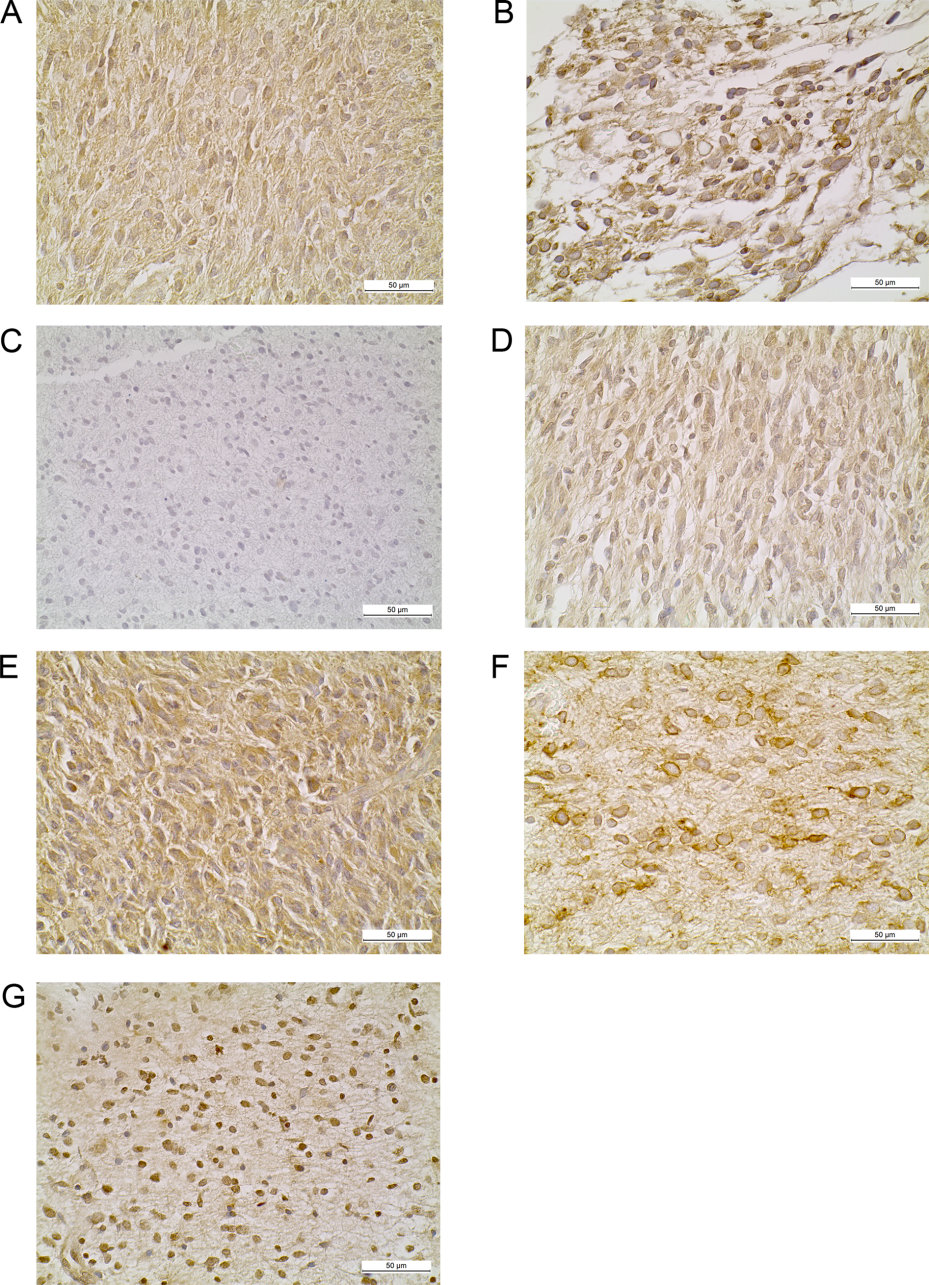
Immunohistochemical studies of the IL-4R signaling axis, *EGFR* and *c-Met* protein expression. Representative immunohistochemical staining of DIPG archival cases for (A) *IL-4* (B) *IL-4Rα* (C) *IL-13* (D) *IL-13Rα1* (E) *IL-13Rα2* (F) *EGFR* (G) *c-Met*.

To interrogate protein-level status of the IL-4 signaling axis in DIPG, we performed IHC analysis of a cohort of formalin fixed paraffin embedded DIPG tumor tissues. Pathologist scoring is detailed in the methods; briefly, 0 = no labeling, 1 = < 10% positive labeling of tumor cells, 2 = 10–50% positive labeling of tumor cells, 3 = > 50% positive labeling of tumor cells. The labeling of tumor cells, when present, was also visually quantified as *weak*, *medium*, *or strong*. Samples not stained for a specific antibody are listed as *NP* (not performed). For *IL-4*, 12/17 (70.5%) cases scored above 0 (Fig 2A). In 6/12 (50.0%) positive cases the expression was medium or strong, and 9/12 (75.0%) were scored as 2 or 3; 2/17 cases (11.8%) scored 0 and 3/17 (17.6%) were not performed (NP). For *IL-4Rα*, 12/17 (70.5%) cases scored above 0 (Fig 2B). In 5/12 (41.6%) cases the expression was strong, and 9/12 (75.0%) were scored as 2 or 3; 3/17 (17.6%) cases scored 0 and 2/17 (11.8%) were NP. For *IL-13*, 1/13 (7.69%) cases scored above 0 (Fig 2C). The positive case had weak labeling with a score of 2; 13/17 (76.5%) cases scored 0 and 3/17 (17.6%) were NP. In many cases scored as 2 or 3, 50% or more of the cells were labeled with different markers. While not all cells were labeled, a significant percentage were; from an immunotherapy perspective, a significant number of tumor cells would be targeted.

The most significant findings were for *IL-13Rα1/2*. For *IL-13Rα1*, 13/17 (76.5%) cases scored above 0 (Fig 2D). All 13 positive cases had weak or medium expression; of these cases, 5/13 (38.5%) had medium expression; 10/13 (76.9%) positive cases had scores of 2 or 3; 3/17 (17.6%) cases scored 0. 1/17 (5.88%) were NP. For *IL-13Rα2*, 12/17 (70.5%) cases scored above 0 (Fig 2E) and 6/12 (50.0%) positive cases had medium or strong expression, all with a score of 2 or 3; 4/17 (23.5%) cases had no labeling and 1/17 (5.88%) were NP.

For reference, we have provided several normal tissue images stained with the different IHC stains, as well as several scored experimental IHC samples (S3 and S4 Figs, respectively).

### Correlation of *IL-4R* staining with other receptors

For *EGFR*, 7/17 (41.1%) cases scored above 0 for *EGFR* and *IL-4Rα* (Fig 2F); 5/7 (71.4%) positive cases had medium or strong expression with a score of 2 or 3; 8/17 cases scored 0 and 2/17 (11.8%) were NP. For *c-Met*, 7/17 (41.1%) cases scored above 0 (Fig 2G). Only 3/7 (42.8%) positive cases were scored as 2 or 3 and 3/7 (42.8%) positive cases had medium or strong labeling, 7/17 (41.2%) cases scored 0 and 3/17 (17.6%) were NP. Table 3 represents correlation of *c-Met* expression with *IL-4* signaling axis genes.

**Table 3:** MET correlation with IL-4 signaling axis genes.

	*c-MET* vs. *IL-4*	*c-MET* vs. *IL-4R*	*c-MET* vs. *IL-13*	*c-MET vs*. *IL-13Ra1*	*c-MET vs*. *IL-13Ra2*
R squared	0.0269	0.5387	0.0642	0.2836	0.0006
P (two-tailed)	0.4135	**< 0.0001**	0.2022	**0.0042**	0.8991
# of paired samples	27	27	27	27	27

## Discussion

Previous studies have reported that *IL-4* blockade exhibits antitumor activity in rodent models of glioma. Follow-up reports elucidated the role of *IL-4* and *IL-13Rα2* as mediators of aberrant Stat3 signaling driving increased expression of anti-apoptotic genes[38, 39]. *IL-13Rα1* and *IL-13Rα2* are known tumor-associated antigens and recent clinical trials have explored inoculation with *IL-13Rα2* and other tumor associated antigens as an immunotherapy[40]. However, the roles of *IL-4* and *IL-4Rα* have not been investigated in DIPG. To this end, we sought to characterize expression of the extended targets of the *IL-4*/*IL-13* signaling axis in DIPG tissues. Of note, exome sequencing did not identify any somatic point mutations or copy number variations in *IL-4*/*IL-13* signaling axis genes. However, consistent with previous reports, RNA-seq gene expression studies revealed significant upregulation of *IL-13Rα1* and *IL-13Rα2* but no statistically significant differential of *IL-4Rα*. Immunohistochemistry revealed a high content of *IL-4*, but not *IL-13*. However, the receptors for these cytokines (*IL-4Rα*, *IL-13Rα1* and *IL-13Rα2*) were all expressed in 85% of the cases analyzed. In 41%, expression of these receptors were all labeled as strong and/or the number cells expressing these receptors was more than 25% of the tumor cell population. These results show that *IL-4* and *IL-13* receptor upregulation is prevalent in DIPG tissues which may be driven by global dysregulation of gene expression mediated by epigenetic mechanisms. Therefore, the potential exists for therapeutic drug targeting of these receptors. The oncogenes *EGFR* and *c-MET* were co-expressed in approximately 41% of these gliomas. Additionally, significant correlations with *IL-13Rα2* (*EGFR*) and *IL-4Rα* and *IL-13Rα1* (c-MET), respectively. These results raise the possibility that *EGFR* may be an epitope worth exploring in combination with *IL-13Rα2* immunotherapies. Previous reports have identified the expression of epidermal growth factor variant III (*EGFRvIII*) in a subset of DIPG tissues with a vaccine pilot study ongoing (NCT01058850)[41, 42]. While we did not examine these tissues for expression of *EGFRvIII*, we found no significant difference in *EGFR* gene expression in tumor tissues versus matched normal and 41% of cases surveyed expressed *EGFR* at the protein level. However, recent sequencing efforts in larger sample sizes have shown that recurrent *EGFR* mutations are infrequent[43]. While the *EGFR* inhibitors erlotinib and gefitinib have demonstrated activity in this disease, this activity may be due in part to off-target kinase inhibition[44, 45]. In summary, we have identified *IL-4Rα*, *IL-13Rα1* and especially *IL-13Rα2* as potential therapeutic targets in DIPG. The co-expression of these receptors with *EGFR* and *c-MET* may further expand immunotherapy options for this disease.

## Materials and methods

### Patient selection for sequencing experiments

We obtained tumor tissues from sixteen (16) new DIPG samples for exome sequencing. Eight (8) samples were provided by Dr. Xiao-Nan Li of Texas Children's Cancer and Hematology Center, four (4) were obtained from OHSU patients, one (1) was obtained from a cell line provided by Dr. Michelle Monje at Stanford University, and three (3) samples were provided by Dr. Esther Hulleman at Vrije Universiteit University Medical Center.

We also obtained tumor tissues from seventeen (17) new DIPG samples for transcriptome sequencing. Nine (8) samples were provided by Dr. Xiao-Nan Li, four (4) were obtained from OHSU patients, one (1) was obtained from a cell line provided by Dr. Michelle Monje, and four (4) samples were provided by Dr. Esther Hulleman. All studies with human tissue were performed with approval of the Oregon Health and Science University (OHSU) institutional review board. Expanded patient data is available in S3 Table.

### Patient selection for immunohistochemistry experiments

We obtained tumor tissues from six (6) autopsy cases at OHSU, and from two (2) patients that underwent a surgical biopsy of their DIPG tumor at OHSU. Eight (8) samples with matched normal brain tissue samples were provided by collaborator Dr. Xiao-Nan Li of Texas Children's Cancer and Hematology Center. Nine (9) additional cases were provided by Dr. Cynthia Hawkins of Toronto Sick Children’s Hospital, in the form of a paraffin block tissue microarray, with an accompanying legend for diagnostic classification of the tissues on the microarray grid. All internal and external cases were morphologically re-evaluated to ensure sample adequacy and diagnostic accuracy. All studies with human tissue were performed with approval of the Oregon Health and Science University (OHSU) institutional review board. Additional information is available in S4 Table.

### RNA-seq

RNA-seq libraries were constructed using the standard TruSeq protocol (Illumina). Briefly, poly(A)+ RNA was isolated from total RNA using oligo-dT attached to magnetic beads. The poly(A)+ was chemically fragmented. Double stranded cDNA was generated using random hexamers as primers for both strands. The cDNAs were blunted-end, then a single A was attached to the 3’ end of each strand to promote ligation. Standard Illumina adapters with indices were ligated to the cDNAs. The resulting ligations were amplified using a limited number of rounds of polymerase chain reaction (PCR). Libraries were separated from unincorporated material using AmpPure beads (Beckman Coulter). Libraries were evaluated using the Bioanalyzer (Agilent) and the concentration of each library was determined by real time PCR (Kapa Biosystems) on a StepOnePlus real time PCR machine (Life Technologies). Sequencing was performed on either a HiSeq 2000 (Illumina) or HiSeq 2500 (Illumina). Sequence assembly was performed using either CASAVA (Illumina) or Bcl2Fastq (Illumina).

### Somatic point mutation identification by exome capture sequencing

The methodology described below is a minor modification of the approach described in[46]. All captured DNA libraries were sequenced with the Illumina HiSeq in paired- end mode, yielding 80 base pairs from the final library fragments. The reads that passed the chastity filter of Illumina BaseCall software were used for subsequent analysis. Matepairs were pooled and mapped as single reads to the reference human genome (NCBI build 36.1, hg18), excluding unordered sequence and alternate haplotypes, using Bowtie[47], keeping unique best hits, and allowing up to two mismatched bases per read. Likely PCR duplicates, defined as reads with equal match intervals on the reference genome, were removed; and individual basecalls with Phred quality less than Q20 were excluded. A mismatched base (SNV) was identified as a variant when 1) it had at least six reads of support, 2) it constituted at least 10% of the coverage at that position, 3) it was observed on both strands, and 4) it fell within 50 bases of a region targeted for capture.

In matched samples (samples with matched normals), a variant was called somatic if 1) there was 8× coverage in the matched normal, 2) it did not occur in the matched normal sample in more than two reads and 2% of the coverage (or 4% of the coverage if the tumor variant fraction was at least 20%), 3) if it had any support in the matched normal, and 4) it was not present in either dbSNP (v137) or the Exome Sequencing Project data set (ESP6500). In unmatched samples, a variant was called probable somatic if it affected the same codon as either a somatic point mutation observed in the matched samples or a somatic mutation from COSMIC (v66). Finally, all somatic/probable somatic variants were screened against the full set of benign samples. Variants were removed from further consideration if they appeared in any benign sample with at least three reads and 10% of the coverage.

### Sanger sequencing

Genomic DNA was isolated from tissue, using the QIAamp DNA mini isolation kit (Qiagen, Valencia, CA, USA), and the sequence of interest was amplified by PCR. Primers were described by Wu et al.[37]. PCR products were subsequently sequenced by the dideoxy chain-termination method, using the ABI PrismTM BigDye Terminator kit (Perkin Elmer, Foster City, CA, USA), run on the ABI Prism Genetic Analyser 3100 automatic DNA autosequencer (Perkin Elmer), and analysed with ABI sequence Alignment Editor software.

### Annotation

We annotated the resulting somatic mutations using CCDS transcripts wherever possible. If no CCDS transcript was available, we use the coding regions of RefSeq transcripts. HUGO gene names were used.

### Exome copy number analysis

Copy number aberrations were quantified and reported for each gene as the segmented normalized log2-transformed exon coverage ratios between each tumor sample and its matched normal as described in[48]. For unmatched samples, we used the average coverage derived from the full set of benign samples as a ‘normal pool;’ for chromosomes X and Y only, we formed separate normal pools from the XX and XY benign samples. We identified segments as focal if they contained 10 or fewer CCDS gene annotations. To identify a gene as gained or lost we first considered the segmented data, requiring a copy number gain or loss of 30% (ratio > = 1.3 or < = 0.7) to call the gene as gained or lost. In addition, we considered the distribution of copy number estimates for each gene’s individual exons. If the mean exonic copy number showed a gain or loss of at least 30% and deviated from the null hypothesis by at least 2.5 s.d., we called the gene gained or lost.

Exome sequencing is available through dbGaP (phs001526.v1.p1).

### Gene expression in RPKM inferred from RNA-seq data

All transcriptome libraries were sequenced with the Illumina HiSeq in paired end mode. The reads that passed the chastity filter of Illumina BaseCall software were used for subsequent analysis. We trimmed all reads to 85-mers and aligned them to the reference human genome (NCBI build 36.1, hg18), plus a splice junction set including 84 bases on either side of the 2008 Illumina splice junction set, using Bowtie[47] in single read mode, keeping unique best hits and allowing up to two mismatched bases. Matepairs from paired-end runs were pooled and treated as single reads.

Next, all of the exons for a single gene were concatenated to form a single ‘transcript’ for that gene. Reads that mapped to the exons in the gene, as well as reads that mapped to the splice junctions, were remapped to the transcript. We then walked the transcript and summed the coverage at each position, then divided the result by the transcript length × the number of reads in the sample, and then multiplied the result by one million. This method is a modified version of the one described in[49].

RNA sequencing is available through dbGaP (phs001526.v1.p1).

### DNA fingerprinting using common high-frequency variants

We considered 147 genomic positions that tended to have good coverage in both whole-exome and RNA-seq data and for which at least two alleles were widespread. For each sample, we constructed a ‘fingerprint’ for those positions with at least six fragments of coverage; an allele was included in the fingerprint if it was seen in at least two fragments of coverage and at least 15% of the total fragments. Two samples were deemed a match if at least 85% of the positions with sufficient coverage in both samples had identical fingerprints. In practice, samples from the same patient matched at more than 90% of positions, whereas samples from different patients matched at fewer than 75% of positions. For this study, all the included samples from the same patient met the 85% match criteria, including all RNA-seq and exome samples. In addition, no samples from different patients exceeded 74% similarity.

### Statistical analyses

Bioinformatics and computational methods are described above. For comparison of gene expression levels of the 429 genes associated with the targets of the drug screen, we generated RNA-seq data and then compared tumor tissues to normal tissues to generate significance scores using a *t*-test with the Benjamini–Hochberg multiple comparison correction.

GraphPad Prism (GraphPad Software Inc., San Diego, CA, USA) was used for all other statistical analyses. Correlation studies were performed using the Pearson product-moment correlation coefficients for select *IL-4* signaling axis genes and the oncogenes *c-Met* and *EGFR*. Comparisons between groups were determined by Student’s t-test. Differences were considered statistically significant when p < 0.05.

### Immunohistochemistry

We performed immunohistochemistry using the following primary antibodies, using the manufacturer’s suggested concentrations: Interleukin 4 (*IL-4*) (Invitrogen, Fredrickson, MD), Interleukin 4 Receptor Alpha (*IL-4Rα*) (RD Systems, Minneapolis, MS), Interleukin 13 (*IL-13*) (Invitrogen), Interleukin 13 Receptor Alpha-1 (*IL-13Rα1*) (RD Systems), Interleukin 13 Receptor Alpha-2 (*IL-13Rα2*) (R&D systems), *MET* Oncogene (Hepatocyte Growth Factor Receptor) (Santa Cruz Biotech, Dallas, TX), Epithelial Growth Factor Receptor (*EGFR*) (Dako, Danvers, MA). Each antibody was first tested with available known positive control normal tissues and tumors, and titrated to obtain an optimal dilution for the best signal/noise ratio. Antigen retrieval procedures were also tested for each antibody, starting with the manufacturer’s recommended reagents. The immunohistochemical staining results were interpreted by two pathologists (SHG and VZ). We used a semi-quantitative scoring system for analysis: 0: No labeling, 1: Less than 10% of tumor cells labeled, 2: 10–50% of tumor cells labeled, 3: More than 50% of tumor cells labeled. The labeling of tumor cells, when present, was also visually quantified as *weak*, *medium*, *or strong*. In interleukin receptor studies, cytoplasmic or membrane labeling was taken into account as positive labeling, but nuclear labeling was disregarded, after our antigen adsorption assays revealed that the nuclear staining was not specific. Each assay was conducted with several identically processed positive control tissues and negative controls were included in all assays. Each antibody was performed in all cases. In four cases, due to depletion of the tissue block, we were able to test only some of the seven antibodies. Where a specific antibody testing was not performed due to limited tissue availability, the designation not present (NP) was used to indicate that the assay was not performed.

## Supporting information

S1 Fig Landscape of commonly mutated genes in DIPG samples. Mutational landscape of the most frequently mutated gene targets. Genes with at least 7 or more variations of any type were considered frequently mutated (average + 2 standard deviations).
(TIF)

S2 Fig Gene expression of genes frequently mutated in DIPG samples. Gene expression of the most frequently mutated exome genes, defines as genes with at least 7 or more variations of any type. Notable genes that are both frequently amplified and overexpressed in DIPG tumor samples include *NTRK1* (a neuronal pro-survival gene) and *MYC* (a gene commonly overexpressed in cancers).
(TIF)

S3 Fig Control staining images for different immunohistochemistry stains. Staining of normal control tissues using different IHC stains used in IHC experiments. (A) *IL-13Rα1* staining of peripheral nerve ganglion. (B) *IL-13Rα2* staining of testicular germ cells. (C) *IL-4Rα* staining of stomach mucosal glands. (D) *MET* staining of intestinal mucosal glands. (E) *EGFR* staining of skin epidermis.
(TIF)

S4 Fig Sample staining definitions for immunohistochemistry straining experiments. Various stains and different assigned scores (0/1/2/3 and weak/medium/strong) as example IHC results for reference. (A) *IL-13R* staining assigned score 0. (B) *IL-13Rα1* staining assigned score 2/medium. (C) *EGFR* staining assigned score 2/strong. (D) *IL-13Rα1* staining assigned score 3/weak. (E) *IL-4Rα* staining assigned score 3/strong.
(TIF)

S1 Table Mutation landscape of DIPG samples. Gene level mutation and variation status of sequenced DIPG samples.
(XLSX)

S2 Table RNA expression across all samples. Quantified RPKM gene expression from whole transcriptome sequencing experiments on DIPG samples.
(XLSX)

S3 Table Patient and sequencing information. De-identified patient information for analyzed DIPG samples.
(XLSX)

S4 Table IHC selection information. Sample name and origin for DIPG samples selected for IHC analysis.
(XLSX)

## References

[1] Donaldson SS, Laningham F, Fisher PG. Advances toward an understanding of brainstem gliomas. J Clin Oncol. 2006;24(8):1266–72. Epub 2006/03/10. doi: 24/8/1266 [pii] doi: 10.1200/JCO.2005.04.6599 . 16525181 10.1200/JCO.2005.04.6599

[2] Hargrave D, Bartels U, Bouffet E. Diffuse brainstem glioma in children: critical review of clinical trials. The Lancet Oncology. 2006;7(3):241–8. doi: 10.1016/S1470-2045(06)70615-5 . 16510333 10.1016/S1470-2045(06)70615-5

[3] MacDonald TJ, Aguilera D, Kramm CM. Treatment of high-grade glioma in children and adolescents. Neuro-oncology. 2011;13(10):1049–58. doi: 10.1093/neuonc/nor092 ; PubMed Central PMCID: PMC3177659. 21784756 10.1093/neuonc/nor092 PMC3177659

[4] Smith SE, Waller JC, Bingham IA, Jewett DM, Nsouli MS, Mackintosh JJ. A diffuse intrinsic pontine glioma roadmap: guiding research toward a cure. Pediatric blood & cancer. 2014;61(5):765–7. Epub 2014/02/01. doi: 10.1002/pbc.24923 . 24481909 10.1002/pbc.24923

[5] Langmoen IA, Lundar T, Storm-Mathisen I, Lie SO, Hovind KH. Management of pediatric pontine gliomas. Childs Nerv Syst. 1991;7(1):13–5. Epub 1991/02/01. . 2054800 10.1007/BF00263825

[6] Packer RJ, Allen JC, Goldwein JL, Newall J, Zimmerman RA, Priest J, et al Hyperfractionated radiotherapy for children with brainstem gliomas: a pilot study using 7,200 cGy. Ann Neurol. 1990;27(2):167–73. Epub 1990/02/01. doi: 10.1002/ana.410270212 . 2317012 10.1002/ana.410270212

[7] Finlay JL, Zacharoulis S. The treatment of high grade gliomas and diffuse intrinsic pontine tumors of childhood and adolescence: a historical—and futuristic—perspective. JNeurooncol. 2005;75(3):253–66. PubMed PMID: 1910. 16195805 10.1007/s11060-005-6747-7

[8] Jennings MT, Sposto R, Boyett JM, Vezina LG, Holmes E, Berger MS, et al Preradiation chemotherapy in primary high-risk brainstem tumors: phase II study CCG-9941 of the Children's Cancer Group. J Clin Oncol. 2002;20(16):3431–7. Epub 2002/08/15. doi: 10.1200/JCO.2002.04.109 . 12177103 10.1200/JCO.2002.04.109

[9] Finlay JL, August C, Packer R, Zimmerman R, Sutton L, Freid A, et al High-dose multi-agent chemotherapy followed by bone marrow 'rescue' for malignant astrocytomas of childhood and adolescence. J Neurooncol. 1990;9(3):239–48. Epub 1990/12/01. . 1964962 10.1007/BF02341155

[10] Bouffet E, Raquin M, Doz F, Gentet JC, Rodary C, Demeocq F, et al Radiotherapy followed by high dose busulfan and thiotepa: a prospective assessment of high dose chemotherapy in children with diffuse pontine gliomas. Cancer. 2000;88(3):685–92. Epub 2000/01/29. doi: 10.1002/(SICI)1097-0142(20000201)88:3<685::AID-CNCR27>3.0.CO;2-K [pii]. . 10649264 10.1002/(sici)1097-0142(20000201)88:3<685::aid-cncr27>3.0.co;2-k

[11] Dunkel IJ, O'Malley B, Finlay JL. Is there a role for high-dose chemotherapy with stem cell rescue for brain stem tumors of childhood? Pediatr Neurosurg. 1996;24(5):263–6. Epub 1996/01/01. doi: 10.1159/000121049 . 8933570 10.1159/000121049

[12] Cohen KJ, Heideman RL, Zhou T, Holmes EJ, Lavey RS, Bouffet E, et al Temozolomide in the treatment of children with newly diagnosed diffuse intrinsic pontine gliomas: a report from the Children's Oncology Group. Neuro Oncol. 2011;13(4):410–6. Epub 2011/02/25. doi: noq205 [pii] doi: 10.1093/neuonc/noq205 . 21345842 10.1093/neuonc/noq205 PMC3064697

[13] Jalali R, Raut N, Arora B, Gupta T, Dutta D, Munshi A, et al Prospective evaluation of radiotherapy with concurrent and adjuvant temozolomide in children with newly diagnosed diffuse intrinsic pontine glioma. Int J Radiat Oncol Biol Phys. 2010;77(1):113–8. Epub 2009/08/04. doi: S0360-3016(09)00597-5 [pii] doi: 10.1016/j.ijrobp.2009.04.031 . 19647954 10.1016/j.ijrobp.2009.04.031

[14] Geoerger B, Hargrave D, Thomas F, Ndiaye A, Frappaz D, Andreiuolo F, et al Innovative Therapies for Children with Cancer pediatric phase I study of erlotinib in brainstem glioma and relapsing/refractory brain tumors. Neuro-oncology. 2011;13(1):109–18. doi: 10.1093/neuonc/noq141 ; PubMed Central PMCID: PMC3018917. 20974795 10.1093/neuonc/noq141 PMC3018917

[15] Grasso CS, Tang Y, Truffaux N, Berlow NE, Liu L, Debily MA, et al Functionally defined therapeutic targets in diffuse intrinsic pontine glioma. Nature medicine. 2015 doi: 10.1038/nm.3855 . 25939062 10.1038/nm.3855 PMC4862411

[16] Kawakami M, Kawakami K, Takahashi S, Abe M, Puri RK. Analysis of interleukin-13 receptor α2 expression in human pediatric brain tumors. Cancer. 2004;101(5):1036–42. doi: 10.1002/cncr.20470 15329913 10.1002/cncr.20470

[17] Debinski W, Gibo DM, Hulet SW, Connor JR, Gillespie GY. Receptor for interleukin 13 is a marker and therapeutic target for human high-grade gliomas. Clinical cancer research: an official journal of the American Association for Cancer Research. 1999;5(5):985–90. Epub 1999/06/03. . 10353730

[18] Debinski W, Obiri NI, Powers SK, Pastan I, Puri RK. Human glioma cells overexpress receptors for interleukin 13 and are extremely sensitive to a novel chimeric protein composed of interleukin 13 and pseudomonas exotoxin. Clinical cancer research: an official journal of the American Association for Cancer Research. 1995;1(11):1253–8. Epub 1995/11/01. . 9815919

[19] Shimamura T, Royal RE, Kioi M, Nakajima A, Husain SR, Puri RK. Interleukin-4 Cytotoxin Therapy Synergizes with Gemcitabine in a Mouse Model of Pancreatic Ductal Adenocarcinoma. Cancer research. 2007;67(20):9903–12. doi: 10.1158/0008-5472.CAN-06-4558 17942922 10.1158/0008-5472.CAN-06-4558

[20] Suzuki A, Leland P, Joshi BH, Puri RK. Targeting of IL-4 and IL-13 receptors for cancer therapy. Cytokine. 2015;75(1):79–88. Epub 2015/06/20. doi: 10.1016/j.cyto.2015.05.026 . 26088753 10.1016/j.cyto.2015.05.026

[21] Thaci B, Brown CE, Binello E, Werbaneth K, Sampath P, Sengupta S. Significance of interleukin-13 receptor alpha 2-targeted glioblastoma therapy. Neuro-Oncology. 2014;16(10):1304–12. Epub 2014/04/12. doi: 10.1093/neuonc/nou045 ; PubMed Central PMCID: PMCPMC4165413. 24723564 10.1093/neuonc/nou045 PMC4165413

[22] Debinski W. An immune regulatory cytokine receptor and glioblastoma multiforme: an unexpected link. Critical reviews in oncogenesis. 1998;9(3–4):255–68. Epub 1999/04/14. . 10201630

[23] Kunwar S, Prados MD, Chang SM, Berger MS, Lang FF, Piepmeier JM, et al Direct Intracerebral Delivery of Cintredekin Besudotox (IL13-PE38QQR) in Recurrent Malignant Glioma: A Report by the Cintredekin Besudotox Intraparenchymal Study Group. Journal of Clinical Oncology. 2007;25(7):837–44. doi: 10.1200/JCO.2006.08.1117 . 17327604 10.1200/JCO.2006.08.1117

[24] Mardor Y, Last D, Daniels D, Shneor R, Maier SE, Nass D, et al Convection-Enhanced Drug Delivery of Interleukin-4 Pseudomonas Exotoxin (PRX321): Increased Distribution and Magnetic Resonance Monitoring. The Journal of Pharmacology and Experimental Therapeutics. 2009;330(2):520–5. doi: 10.1124/jpet.109.154401 PubMed PMID: PMC3202436. 19478131 10.1124/jpet.109.154401 PMC3202436

[25] Van Mieghem E, Wozniak A, Geussens Y, Menten J, De Vleeschouwer S, Van Calenbergh F, et al Defining pseudoprogression in glioblastoma multiforme. European journal of neurology: the official journal of the European Federation of Neurological Societies. 2013;20(10):1335–41. doi: 10.1111/ene.12192 . 23679051 10.1111/ene.12192

[26] Pollack IF, Jakacki RI, Butterfield LH, Hamilton RL, Panigrahy A, Potter DM, et al Antigen-specific immune responses and clinical outcome after vaccination with glioma-associated antigen peptides and polyinosinic-polycytidylic acid stabilized by lysine and carboxymethylcellulose in children with newly diagnosed malignant brainstem and nonbrainstem gliomas. Journal of clinical oncology: official journal of the American Society of Clinical Oncology. 2014;32(19):2050–8. Epub 2014/06/04. doi: 10.1200/jco.2013.54.0526 ; PubMed Central PMCID: PMCPMC4067943. 24888813 10.1200/JCO.2013.54.0526 PMC4067943

[27] Brown CE, Alizadeh D, Starr R, Weng L, Wagner JR, Naranjo A, et al Regression of Glioblastoma after Chimeric Antigen Receptor T-Cell Therapy. New England Journal of Medicine. 2016;375(26):2561–9. doi: 10.1056/NEJMoa1610497 . 28029927 10.1056/NEJMoa1610497 PMC5390684

[28] Kellner C, Bruenke J, Stieglmaier J, Schwemmlein M, Schwenkert M, Singer H, et al A novel CD19-directed recombinant bispecific antibody derivative with enhanced immune effector functions for human leukemic cells. Journal of immunotherapy. 2008;31(9):871–84. doi: 10.1097/CJI.0b013e318186c8b4 . 18833000 10.1097/CJI.0b013e318186c8b4

[29] Dreier T, Lorenczewski G, Brandl C, Hoffmann P, Syring U, Hanakam F, et al Extremely potent, rapid and costimulation-independent cytotoxic T-cell response against lymphoma cells catalyzed by a single-chain bispecific antibody. International journal of cancer Journal international du cancer. 2002;100(6):690–7. doi: 10.1002/ijc.10557 . 12209608 10.1002/ijc.10557

[30] He Q, Zhang H, Wang Y, Ting HH, Yu W, Cao X, et al Purified anti-CD3 x anti-HER2 bispecific antibody potentiates cytokine-induced killer cells of poor spontaneous cytotoxicity against breast cancer cells. Cell & bioscience. 2014;4(1):70 doi: 10.1186/2045-3701-4-70 ; PubMed Central PMCID: PMC4258008. 25485089 10.1186/2045-3701-4-70 PMC4258008

[31] Mazzoleni S, Politi LS, Pala M, Cominelli M, Franzin A, Sergi Sergi L, et al Epidermal growth factor receptor expression identifies functionally and molecularly distinct tumor-initiating cells in human glioblastoma multiforme and is required for gliomagenesis. Cancer Res. 2010;70(19):7500–13. Epub 2010/09/23. doi: 0008-5472.CAN-10-2353 [pii] doi: 10.1158/0008-5472.CAN-10-2353 . 20858720 10.1158/0008-5472.CAN-10-2353

[32] Paugh BS, Qu C, Jones C, Liu Z, Adamowicz-Brice M, Zhang J, et al Integrated molecular genetic profiling of pediatric high-grade gliomas reveals key differences with the adult disease. J Clin Oncol. 2010;28(18):3061–8. Epub 2010/05/19. doi: JCO.2009.26.7252 [pii] doi: 10.1200/JCO.2009.26.7252 ; PubMed Central PMCID: PMC2903336. 20479398 10.1200/JCO.2009.26.7252 PMC2903336

[33] Yano H, Hara A, Murase S, Hayashi K, Ando H, Shinoda J, et al Expression of hepatocyte growth factor and matrix metalloproteinase-2 in human glioma. Brain Tumor Pathol. 2001;18(1):7–12. Epub 2001/08/24. . 11517976 10.1007/BF02478919

[34] Hamasuna R, Kataoka H, Meng JY, Itoh H, Moriyama T, Wakisaka S, et al Reduced expression of hepatocyte growth factor activator inhibitor type-2/placental bikunin (HAI-2/PB) in human glioblastomas: implication for anti-invasive role of HAI-2/PB in glioblastoma cells. Int J Cancer. 2001;93(3):339–45. Epub 2001/07/04. doi: 10.1002/ijc.1349 [pii]. . 11433397 10.1002/ijc.1349

[35] Paugh BS, Broniscer A, Qu C, Miller CP, Zhang J, Tatevossian RG, et al Genome-wide analyses identify recurrent amplifications of receptor tyrosine kinases and cell-cycle regulatory genes in diffuse intrinsic pontine glioma. J Clin Oncol. 2011;29(30):3999–4006. Epub 2011/09/21. doi: JCO.2011.35.5677 [pii] doi: 10.1200/JCO.2011.35.5677 . 21931021 10.1200/JCO.2011.35.5677 PMC3209696

[36] Karajannis M, Allen JC, Newcomb EW. Treatment of Pediatric Brain Tumors. Journal of cellular physiology. 2008;217(3):584–9. doi: 10.1002/jcp.21544 PubMed PMID: PMC2574972. 18651562 10.1002/jcp.21544 PMC2574972

[37] Wu G, Broniscer A, McEachron TA, Lu C, Paugh BS, Becksfort J, et al Somatic histone H3 alterations in pediatric diffuse intrinsic pontine gliomas and non-brainstem glioblastomas. Nat Genet. 2012;44(3):251–3. Epub 2012/01/31. doi: 10.1038/ng.1102 ; PubMed Central PMCID: PMCPmc3288377. 22286216 10.1038/ng.1102 PMC3288377

[38] Benedetti S, Pirola B, Pollo B, Magrassi L, Bruzzone MG, Rigamonti D, et al Gene therapy of experimental brain tumors using neural progenitor cells. Nat Med. 2000;6(4):447–50. Epub 2000/03/31. doi: 10.1038/74710 . 10742153 10.1038/74710

[39] Rahaman SO, Vogelbaum MA, Haque SJ. Aberrant Stat3 signaling by interleukin-4 in malignant glioma cells: involvement of IL-13Ralpha2. Cancer research. 2005;65(7):2956–63. doi: 10.1158/0008-5472.CAN-04-3592 . 15805299 10.1158/0008-5472.CAN-04-3592

[40] Joshi BH, Plautz GE, Puri RK. Interleukin-13 receptor alpha chain: a novel tumor-associated transmembrane protein in primary explants of human malignant gliomas. Cancer research. 2000;60(5):1168–72. . 10728667

[41] Bax DA, Gaspar N, Little SE, Marshall L, Perryman L, Regairaz M, et al EGFRvIII deletion mutations in pediatric high-grade glioma and response to targeted therapy in pediatric glioma cell lines. Clinical cancer research: an official journal of the American Association for Cancer Research. 2009;15(18):5753–61. doi: 10.1158/1078-0432.CCR-08-3210 . 19737945 10.1158/1078-0432.CCR-08-3210

[42] Li G, Mitra SS, Monje M, Henrich KN, Bangs CD, Nitta RT, et al Expression of epidermal growth factor variant III (EGFRvIII) in pediatric diffuse intrinsic pontine gliomas. Journal of neuro-oncology. 2012;108(3):395–402. doi: 10.1007/s11060-012-0842-3 ; PubMed Central PMCID: PMC3368992. 22382786 10.1007/s11060-012-0842-3 PMC3368992

[43] Wu G, Diaz AK, Paugh BS, Rankin SL, Ju B, Li Y, et al The genomic landscape of diffuse intrinsic pontine glioma and pediatric non-brainstem high-grade glioma. Nature genetics. 2014;46(5):444–50. doi: 10.1038/ng.2938 ; PubMed Central PMCID: PMC4056452. 24705251 10.1038/ng.2938 PMC4056452

[44] Conradt L, Godl K, Schaab C, Tebbe A, Eser S, Diersch S, et al Disclosure of erlotinib as a multikinase inhibitor in pancreatic ductal adenocarcinoma. Neoplasia. 2011;13(11):1026–34. ; PubMed Central PMCID: PMC3223607. 22131878 10.1593/neo.111016 PMC3223607

[45] Brehmer D, Greff Z, Godl K, Blencke S, Kurtenbach A, Weber M, et al Cellular targets of gefitinib. Cancer research. 2005;65(2):379–82. . 15695376

[46] Grasso CS, Wu YM, Robinson DR, Cao X, Dhanasekaran SM, Khan AP, et al The mutational landscape of lethal castration-resistant prostate cancer. Nature. 2012;487(7406):239–43. Epub 2012/06/23. doi: 10.1038/nature11125 ; PubMed Central PMCID: PMCPmc3396711. 22722839 10.1038/nature11125 PMC3396711

[47] Langmead B, Trapnell C, Pop M, Salzberg S. Ultrafast and memory-efficient alignment of short DNA sequences to the human genome. Genome Biology. 2009;10(3):R25 doi: 10.1186/gb-2009-10-3-r25 19261174 10.1186/gb-2009-10-3-r25 PMC2690996

[48] Lonigro RJ, Grasso CS, Robinson DR, Jing X, Wu Y-M, Cao X, et al Detection of Somatic Copy Number Alterations in Cancer Using Targeted Exome Capture Sequencing. Neoplasia (New York, NY). 2011;13(11):1019–25. PubMed PMID: PMC3223606. 10.1593/neo.111252 PMC3223606 22131877

[49] Mortazavi A, Williams BA, McCue K, Schaeffer L, Wold B. Mapping and quantifying mammalian transcriptomes by RNA-Seq. Nat Meth. 2008;5(7):621–8. http://www.nature.com/nmeth/journal/v5/n7/suppinfo/nmeth.1226_S1.html. 10.1038/nmeth.1226 PMC13303166 18516045

