# Supplementary figures and images for: IL-13 receptors as possible therapeutic targets in diffuse intrinsic pontine glioma
Source: PLoS One. 2018 Apr 5;13(4):e0193565. doi: 10.1371/journal.pone.0193565 (PMC5886401; doi:10.1371/journal.pone.0193565)

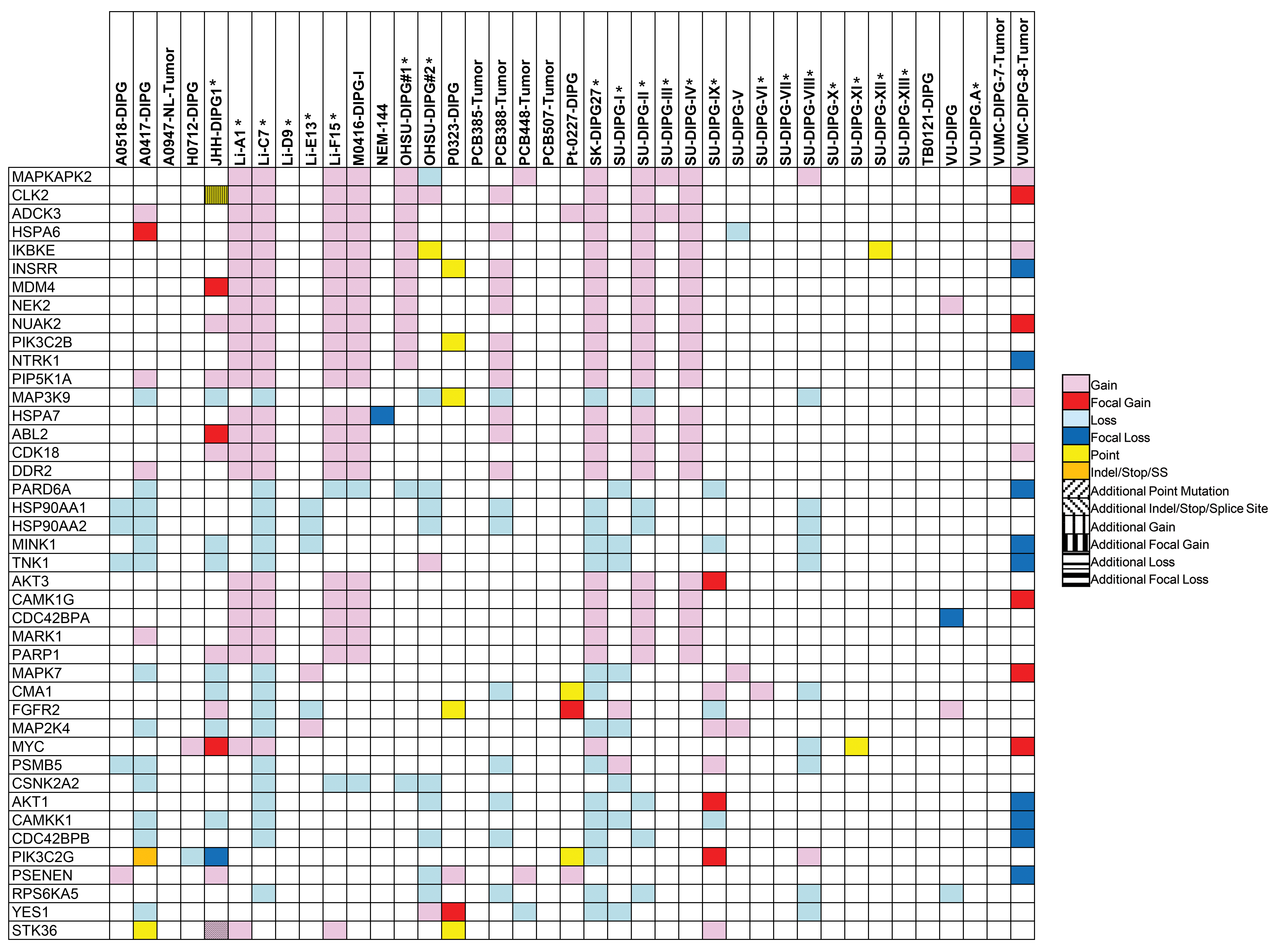

Supplement: S1 Fig — Mutational landscape of the most frequently mutated gene targets. Genes with at least 7 or more variations of any type were considered frequently mutated (average + 2 standard deviations). (TIF) [file pone.0193565.s001.tif]

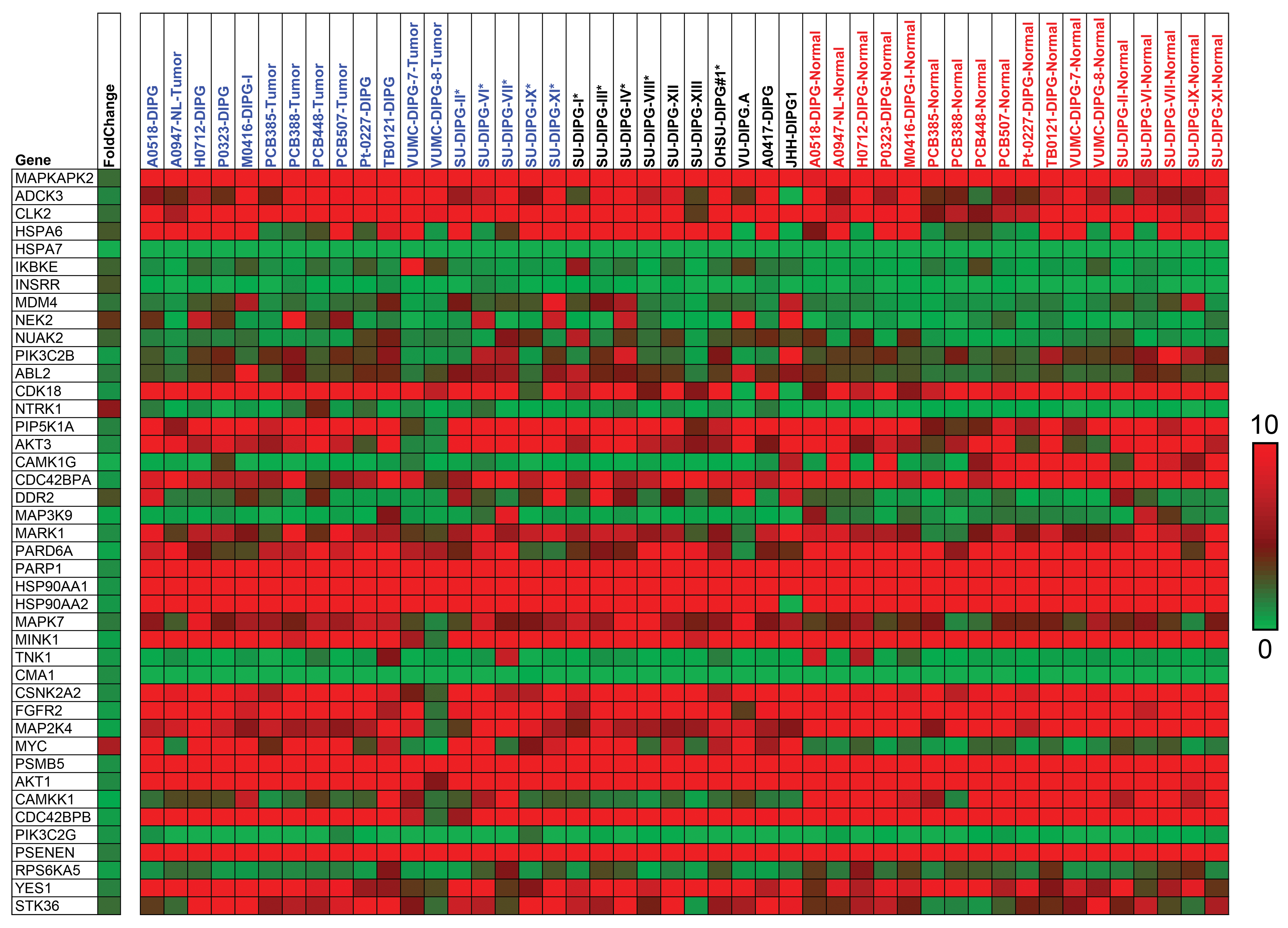

Supplement: S2 Fig — Gene expression of the most frequently mutated exome genes, defines as genes with at least 7 or more variations of any type. Notable genes that are both frequently amplified and overexpressed in DIPG tumor samples include NTRK1 (a neuronal pro-survival gene) and MYC (a gene commonly overexpressed in cancers). (TIF) [file pone.0193565.s002.tif]

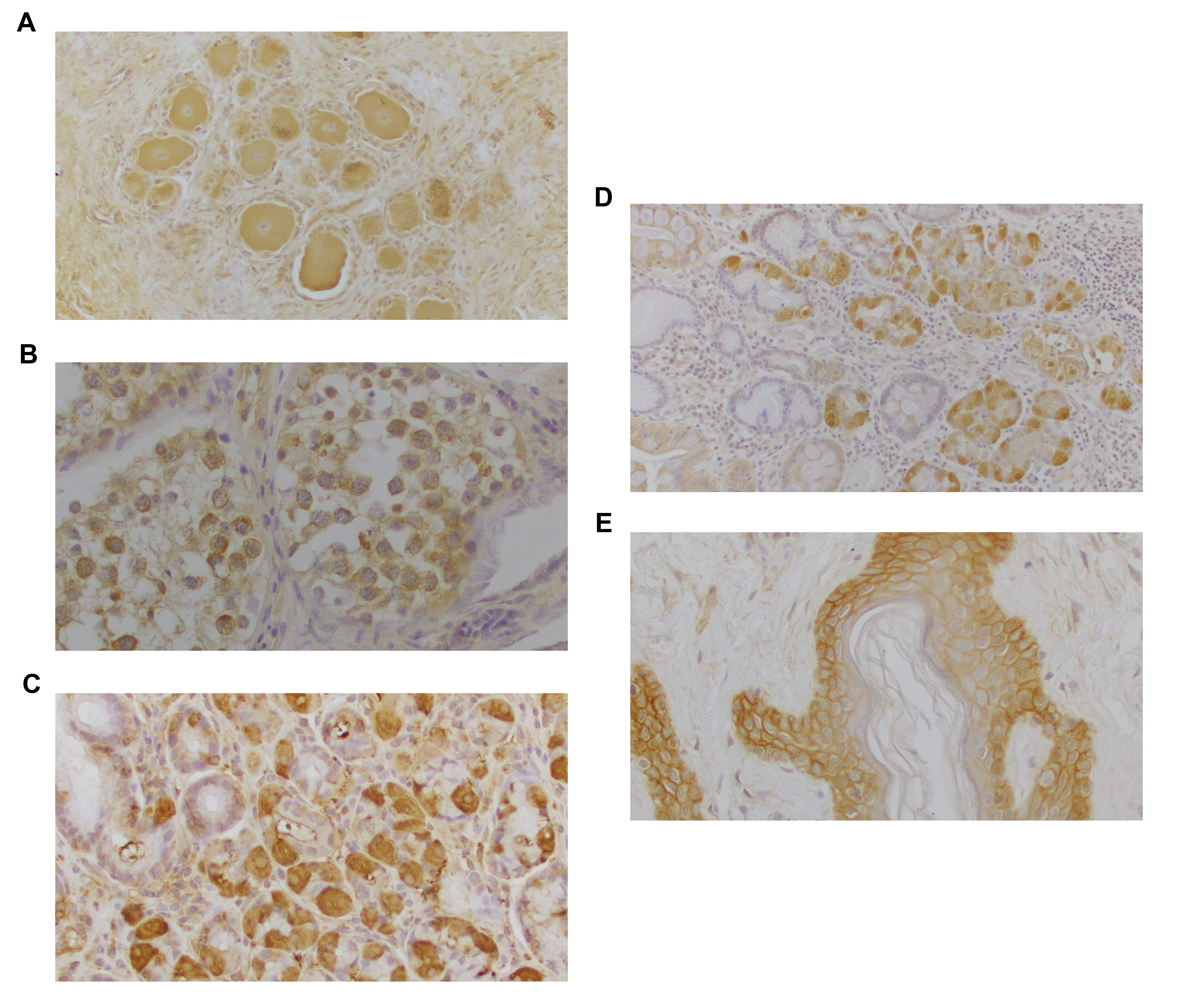

Supplement: S3 Fig — Staining of normal control tissues using different IHC stains used in IHC experiments. (A) IL-13Rα1 staining of peripheral nerve ganglion. (B) IL-13Rα2 staining of testicular germ cells. (C) IL-4Rα staining of stomach mucosal glands. (D) MET staining of intestinal mucosal glands. (E) EGFR staining of skin epidermis. (TIF) [file pone.0193565.s003.tif]

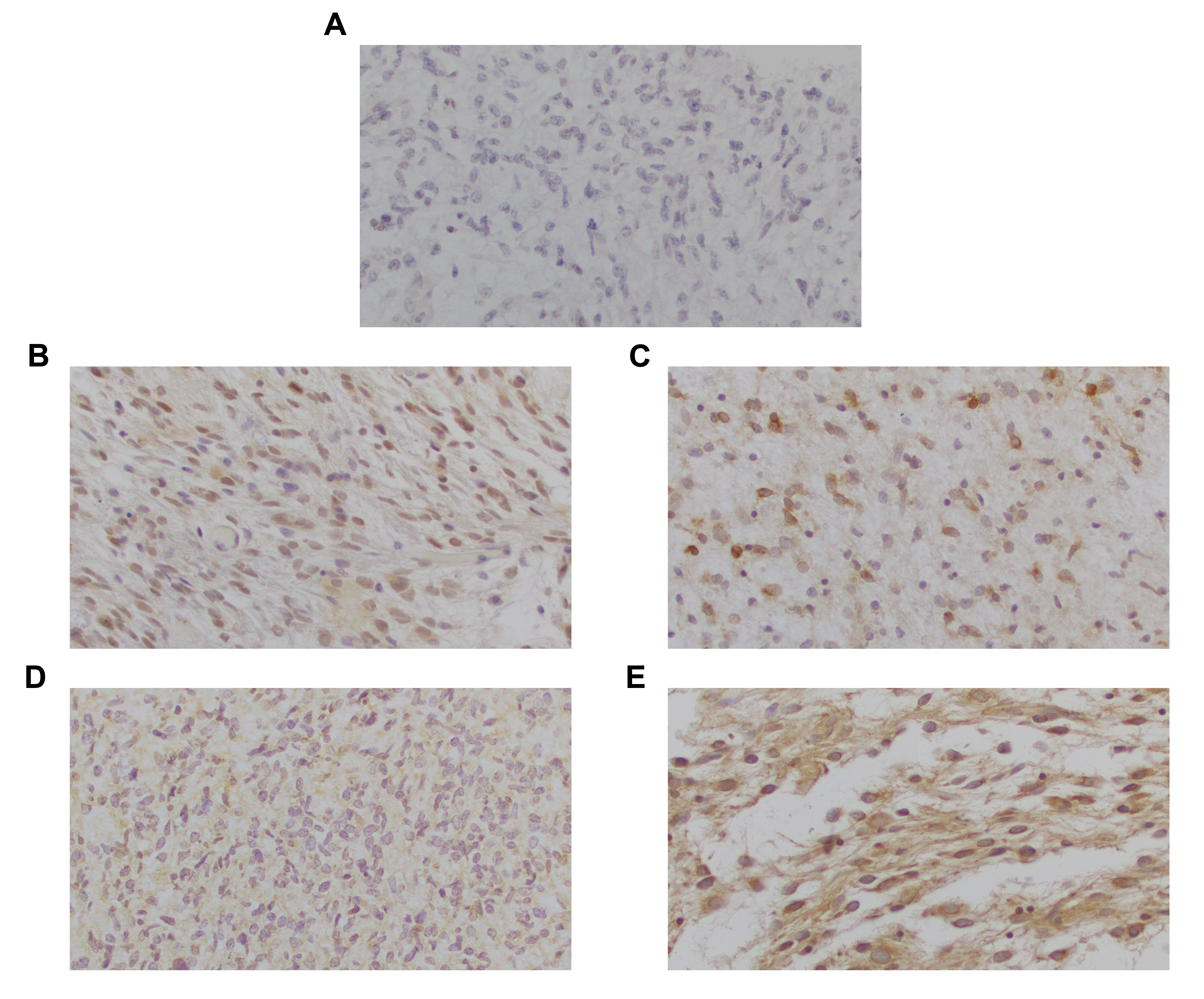

Supplement: S4 Fig — Various stains and different assigned scores (0/1/2/3 and weak/medium/strong) as example IHC results for reference. (A) IL-13R staining assigned score 0. (B) IL-13Rα1 staining assigned score 2/medium. (C) EGFR staining assigned score 2/strong. (D) IL-13Rα1 staining assigned score 3/weak. (E) IL-4Rα staining assigned score 3/strong. (TIF) [file pone.0193565.s004.tif]
